# The PRESIDE (PhaRmacogEnomicS In DEpression) Trial: a double-blind randomised controlled trial of pharmacogenomic-informed prescribing of antidepressants on depression outcomes in patients with major depressive disorder in primary care

**DOI:** 10.1186/s13063-023-07361-6

**Published:** 2023-05-19

**Authors:** Sibel Saya, Patty Chondros, Anastasia Abela, Cathrine Mihalopolous, Mary Lou Chatterton, Jane Gunn, Timothy F. Chen, Thomas M. Polasek, Elise Dettmann, Rachel Brooks, Michelle King, Luke Spencer, Pavithran Alphonse, Shakira Milton, Georgia Ramsay, Zoe Siviour, Jamie Liew, Philip Ly, Matthew Thoenig, Raushaan Seychell, Floriana La Rocca, Luke B. Hesson, Nydia Mejias, Terri Sivertsen, Melanie Anne Galea, Chad Bousman, Jon Emery

**Affiliations:** 1 Department of General Practice and Primary Care, Melbourne Medical School, University of Melbourne, Melbourne, Australia; 2 Centre for Cancer Research, University of Melbourne, Melbourne, Australia; 3 School of Public Health and Preventive Medicine, Monash University Health Economics Group, Monash University, Melbourne, VIC Australia; 4 Sydney Pharmacy School, Faculty of Medicine and Health, The University of Sydney, Camperdown, NSW 2006 Australia; 5 Certara, Princeton, NJ USA; 6 Centre for Medicine Use and Safety, Monash University, Melbourne, Australia; 7 Genetics Department, Douglass Hanly Moir Pathology, Sonic Healthcare, Sydney, NSW Australia; 8 School of Clinical Medicine, Faculty of Medicine, UNSW Sydney, Randwick, NSW Australia; 9 Kinghorn Centre for Clinical Genomics, Garvan Institute of Medical Research, Darlinghurst, NSW Australia; 10 Translational Software, Mercer Island, WA USA; 11 Department of Medical Genetics, University of Calgary, Calgary, AB Canada

**Keywords:** Major depressive disorder, Mental health, Pharmacogenomics, Primary care, General practice, Antidepressants, Randomised controlled trial

## Abstract

**Background:**

The evidence for the clinical utility of pharmacogenomic (PGx) testing is growing, and guidelines exist for the use of PGx testing to inform prescribing of 13 antidepressants. Although previous randomised controlled trials of PGx testing for antidepressant prescribing have shown an association with remission of depression in clinical psychiatric settings, few trials have focused on the primary care setting, where most antidepressant prescribing occurs.

**Methods:**

The PRESIDE Trial is a stratified double-blinded randomised controlled superiority trial that aims to evaluate the impact of a PGx-informed antidepressant prescribing report (compared with standard prescribing using the Australian Therapeutic Guidelines) on depressive symptoms after 12 weeks, when delivered in primary care. Six hundred seventy-two patients aged 18–65 years of general practitioners (GPs) in Victoria with moderate to severe depressive symptoms, measured using the Patient Health Questionnaire-9 (PHQ-9), will be randomly allocated 1:1 to each arm using a computer-generated sequence. Participants and GPs will be blinded to the study arm. The primary outcome is a difference between arms in the change of depressive symptoms, measured using the PHQ-9 after 12 weeks. Secondary outcomes include a difference between the arms in change in PHQ-9 score at 4, 8 and 26 weeks, proportion in remission at 12 weeks, a change in side effect profile of antidepressant medications, adherence to antidepressant medications, change in quality of life and cost-effectiveness of the intervention.

**Discussion:**

This trial will provide evidence as to whether PGx-informed antidepressant prescribing is clinically efficacious and cost-effective. It will inform national and international policy and guidelines about the use of PGx to select antidepressants for people with moderate to severe depressive symptoms presenting in primary care.

**Trial registration:**

Australian and New Zealand Clinical Trial Registry ACTRN12621000181808. Registered on 22 February 2021.

## Administrative information

**Table:** 

Title {1}	The PRESIDE (PhaRmacogEnomicS In DEpression) Trial: a double-blind randomised controlled trial of pharmacogenomic-informed prescribing of antidepressants on depression outcomes in patients with major depressive disorder in primary care
Trial registration {2a and 2b}	ANZCTR: ACTRN12621000181808
Protocol version {3}	PRESIDE protocol V1.2 July 2022
Funding {4}	The Medical Research Future Fund (MRFF, ID MRF1200060)
Author details {5a}	Sibel Saya^1,2^, Patty Chondros^1^, Anastasia Abela^1,2^, Cathrine Mihalopolous^3^, Mary Lou Chatterton^3^, Jane Gunn^1^, Timothy F Chen^4^, Thomas M Polasek^5,6^, Elise Dettmann^1^, Rachel Brooks^1,2^, Michelle King^1,2^, Luke Spencer^1,2^, Pavithran Alphonse^1,2^, Shakira Milton^1,2^, Georgia Ramsay^1,2^, Zoe Siviour^1,2^, Jamie Liew^1,2^, Philip Ly^1,2^, Matthew Thoenig^1,2^, Raushaan Seychell^1,2^, Floriana La Rocca^1,2^, Luke B Hesson^7.8.9^, Nydia Mejias^10^, Terri Sivertsen^7^, Melanie Anne Galea^7^, Chad Bousman^11^, Jon Emery^1,2^ 1. Department of General Practice and Primary Care, Melbourne Medical School, University of Melbourne, Melbourne, Australia 2. Centre for Cancer Research, University of Melbourne, Melbourne, Australia 3. Monash University Health Economics Group, School of Public Health and Preventive Medicine, Monash University, Melbourne, VIC, Australia 4. Sydney Pharmacy School, Faculty of Medicine and Health, The University of Sydney, Camperdown, NSW, 2006, Australia 5. Certara, Princeton, NJ, USA 6. Centre for Medicine Use and Safety, Monash University, Melbourne, Australia 7. Genetics Department, Douglass Hanly Moir Pathology, Sonic Healthcare, Sydney, NSW, Australia 8. School of Clinical Medicine, Faculty of Medicine, UNSW Sydney, Randwick, NSW, Australia 9. Kinghorn Centre for Clinical Genomics, Garvan Institute of Medical Research, Darlinghurst, NSW, Australia 10. Translational Software, WA, USA 11. Department of Medical Genetics, University of Calgary, Calgary, Alberta, Canada
Name and contact information for the trial sponsor {5b}	Clinical Trials Governance The University of Melbourne Contact (Trial CI): jon.emery@unimelb.edu.au
Role of sponsor {5c}	The sponsor does not have input or ultimate authority in the study design; collection, management, analysis and interpretation of the data; writing of the report; and the decision to submit the report for publication

## Introduction

### Background and rationale {6a}

#### Prevalence of depression worldwide and in Australia

Depression affects at least 264 million people worldwide and is a leading cause of non-fatal burden of disease [1]. Australia ranks second in the prevalence of depression worldwide [2]. It causes significant costs to individuals and to society through medical costs and loss of productivity [3]. The majority of people with depression are identified, treated and followed up by general practitioners (GPs), managing patients across the spectrum of disease severity [4]. Therefore, interventions to improve the effectiveness and cost-effectiveness of managing depression have the greatest chance of impact when focused on primary care.

#### Treatment of major depressive disorder

Depression, clinically referred to as major depressive disorder (MDD), is primarily treated with a combination of antidepressant medication and psychological interventions [5]. First-line antidepressant medications are selective serotonin reuptake inhibitors (SSRIs) and serotonin-noradrenaline reuptake inhibitors (SNRIs). SSRIs are the most common medications prescribed for MDD as they have fewer reported side effects [6]. Many patients do not respond to these classes of medication or experience intolerable side effects. Up to a half of patients with MDD do not respond to their first antidepressant [7], and remission rates are as low as 37.5% [8]. This leads to prolonged duration of symptoms, increased burden of side-effects for limited benefit and greater medical costs [9]. While the antidepressant response is multifactorial, genetic factors contribute 42% of this variance in drug response [10]. This has led to the development of international pharmacogenomic-based guidelines which use an individual’s genetic information to inform the selection and dosing of antidepressants [11–13].

#### Pharmacogenomic-informed prescribing of antidepressants

Pharmacogenomic (PGx) testing for variants in genes that encode key proteins involved in pharmacokinetics and pharmacodynamics can guide drug and dose selection with the aim of improving efficacy and decreasing adverse effects [14].

To ensure standardised and evidence-based implementation of PGx testing results, the Clinical Pharmacogenetics Implementation Consortium (CPIC) and Royal Dutch Pharmacogenetics Working Group (DPWG) have produced guidelines to inform the use of genotyping for prescribing 14 antidepressant medications, including SSRIs, SNRIs and tricyclic antidepressants (TCA) [11–13]. Recommendations are based on genotype-predicted metaboliser phenotypes of the cytochrome P450 genes *CYP2D6*, *CYP2C19* and/or *CYP2B6*. At the time this trial was designed, these guidelines included recommendations for 13 antidepressants incorporating predicted metaboliser phenotypes of *CYP2D6* and *CYP2C19*.

Previous randomised controlled trials of PGx testing for antidepressant prescribing have shown an almost 50% relative increase in the proportion of remission of major depression compared to usual care (risk ratio = 1.46, 95% CI 1.13–1.88, *p* = 0.003) [15]. However, participants and treating clinicians were not blinded to intervention allocation in these trials, leading to possible information bias. Additionally, most participants were recruited from psychiatric settings, with a minority from primary care, where 86% of antidepressant prescribing occurs [16], limiting the generalisability of results. Finally, studies rarely followed patients beyond 12 weeks and did not measure improvement in long-term depressive symptoms.

Recent primary care data has shown the potential clinical utility of PGx testing for antidepressants in this setting, with 45–84% of prescribed antidepressants in an Australian cohort having an associated pharmacogenetic guideline that could guide dose [17]. Sixty-six per cent had combined *CYP2D6* and *CYP2C19* genotype-predicted metaboliser phenotypes which would be considered actionable by CPIC or DPWG antidepressant prescribing guidelines. Furthermore, one-quarter of patients were taking an antidepressant medication which would not be recommended based on their *CYP2D6* and/or *CYP2C19* genotype-predicted metaboliser phenotype [17].

Although there is significant literature highlighting the utility of *CYP2D6* and *CYP2C19* genotyping in reducing gene-antidepressant mismatches, a knowledge gap exists about its clinical application in primary care. Therefore, the PRESIDE (PhaRmacogEnomicS In DEpression) Trial aims to fill that gap, through a randomised double-blinded controlled trial.

## Objectives {7}

### Primary objective

The primary objective is to determine the efficacy of a PGx-informed antidepressant prescribing report on depressive symptoms at 12 weeks after GP receipt of the prescribing report, when delivered in primary care for patients aged 18 to 65 years old with moderate to severe depressive symptoms, compared to a prescribing report based on the current Australian Therapeutic Guidelines (Psychotropic) for antidepressant prescribing (i.e. standard of care) [5].

### Secondary objectives

The secondary objective is to determine the effect of PGx-informed antidepressant prescribing, compared to the control prescribing report on the following:

- Change in depressive symptoms at 4, 8 and 26 weeks
- Depressive symptom remission at 12 weeks
- Depressive symptom response at 12 weeks
- Side effect frequency at 4, 8, 12 and 26 weeks
- Medication adherence at 4, 8, 12 and 26 weeks
- Quality of life at 4, 8, 12 and 26 weeks
- Number of antidepressant medication changes within 26 weeks
- Cost-effectiveness within 26 weeks

## Trial design {8}

The PRESIDE Trial is a multi-site, double-blinded, individually randomised controlled superiority trial with a 1:1 allocation of participants to the experimental (PGx-informed) and control (Australian TG-informed) prescribing interventions. This protocol is reported in accordance with the SPIRIT (Standard Protocol Items: Recommendations for Interventional Trials) guidance [18].

## Methods: participants, interventions and outcomes

### Study setting {9}

In general practice clinics across Victoria, Australia, practices are recruited from areas representing a broad range of sociodemographic backgrounds to reflect the wider population of Victoria, Australia.

### Eligibility criteria {10}

#### Eligibility criteria for general practice clinics

General practices are approached to participate in the study if they have two or more full-time equivalent GPs to ensure a sufficient volume of potential participants. General practices are excluded if they do not have at least one private room for researchers to conduct recruitment activities. Individual GPs within clinics are consented to the study, allowing for researchers to approach their patients to participate and at least two GPs must consent to be a part of the trial.

#### Inclusion criteria for participants

Participants are eligible if they are:

(i) Aged between 18 and 65 years old, inclusive
(ii) Have an upcoming appointment with a consented GP within 2 days of being approached for participation in the trial
(iii) Score a total of 10 on the Patient Health Questionnaire 9 [19] (PHQ-9) indicating at least moderate depressive symptoms in the past two weeks
(iv) Are able to read and understand English
(v) Are competent to give informed consent

#### Exclusion criteria for participants

Participants are ineligible if they:

(i) Are currently taking antipsychotic medication, except if taking quetiapine ≤ 100 mg PRN for sleep, with no history of psychosis
(ii) Are pregnant
(iii) Report that they have had suicidal thoughts ‘nearly every day’, as per question 9 on the PHQ-9
(iv) Have a current diagnosis of dementia
(v) Have an active diagnosis of COVID-19
(vi) Are unavailable over the next 6 months for study follow-up

The first exclusion criterion was stipulated to omit potential participants who have a history of psychosis, given the additional complexity of the management of depressive symptoms in this group, which often also occurs outside of primary care. The original wording of this exclusion criterion was “currently taking antipsychotic medication”. On 22 July 2022, after the 204th participant was recruited, this was amended to allow low-dose use of quetiapine as several unnecessary exclusions were made of potentially eligible participants who were taking low doses of quetiapine for sleep disturbance, without any history of psychosis. The fifth exclusion criterion was added on 17 November 2021 as a safety precaution for researchers handling the study DNA samples.

### Who will take informed consent? {26a}

#### General practitioner informed consent

Members of the research team provide the study rationale and participant recruitment processes to interested GP clinics then invite discussion about the study. This includes information about PGx testing, its potential utility in guiding antidepressant prescribing and the prescribing reports they will receive for each of their patients recruited to the study. It is emphasised that GPs should use their clinical judgement when discussing and determining what, if any, treatment to commence for their patient’s depressive symptoms. GPs are reminded of the clinical guidelines for the management of depression that state patients should be followed up every 4 weeks. Each GP is provided a GP information sheet about the study, given the opportunity to ask questions and individually consented to the study to allow recruitment of their patients.

#### Patient informed consent for trial participation

Trained research assistants provide individuals who have appointments with consented GPs within 2 days of approach with verbal and written information about the trial, check their eligibility and answer any questions about the study. A second research assistant obtains written informed consent if they agree to participate in the trial. Due to COVID-19 and resulting government restrictions, both face-to-face and teletrial methods are used for approach, eligibility assessment and informed consent discussions with potential participants. Interested and eligible participants are provided with the study information sheet prior to consenting to the study and given the opportunity to ask questions. Participants recruited via teletrial complete an online e-consent form through the study’s REDCap database [20]. A copy (either hard or electronic) of their study consent form is provided to participants.

#### Patient informed consent for release of administrative health service use and prescribing data (optional)

Additional and optional written consent is also sought for the release of participants’ Medicare Benefits Schedule (MBS) and Pharmaceutical Benefits Scheme (PBS) data via Services Australia. Initial approvals for the collection of administrative data on health service use and prescription medication dispensing from our government-funded health service (administered by Services Australia) were substantially delayed. Therefore, it was decided by the study steering committee that recruitment for the trial should begin prior to this approval being provided. Upon approval, these participants were retrospectively contacted to obtain this consent. Furthermore, participants not entitled to government-funded healthcare (e.g. they are foreign citizens and not permanent residents of Australia) do not have any Services Australia data available; however, we are collecting additional data directly from the GP record of participants, as well as self-reported use of health services and medications.

### Additional consent provisions for collection and use of participant data and biological specimens {26b}

Participants give specific consent for the study, in that their data and biological sample will not be used for future studies. Any excess DNA is securely disposed of by the laboratory conducting the PGx test (Sonic Healthcare, Sydney, Australia). If a sample fails to yield a result, the sample is tested once more before it is securely discarded.

## Interventions

### Explanation for the choice of comparators {6b}

Participants are randomised to receive either PGx-informed prescribing (experimental intervention) or Australian Therapeutic Guidelines-informed prescribing (control intervention). The sole difference between the experimental and control interventions is the method used to make dosing recommendations in the report the GP receives. The dosing recommendations offered in the control intervention are based on the Australian Therapeutic Guidelines, whereas the dosing recommendations offered in the experimental intervention are based on the participant’s *CYP2D6* and *CYP2C19* genotype-predicted metaboliser phenotypes.

Previous clinical trials of PGx-informed antidepressant prescribing have exclusively used treatment as usual (i.e. standard prescribing) as the comparator [15]. However, this comparator does not allow blinding of the treating clinician and results in a higher risk of performance bias as well as attention and ancillary treatment biases. As such, the comparator in the PRESIDE Trial is Australian Therapeutic Guideline-informed prescribing, delivered using a prescribing report that is formatted identically to the experimental intervention.

### Intervention description {11a}

#### Determination of PGx genotype and phenotype 

Prior to randomisation, all participants provide a saliva sample using the ORAcollect®-DNA OCR100 kit (DNA Genotek, Ottawa, Canada). Saliva samples are sent via Melbourne Pathology to Douglass Hanly Moir Pathology (Sonic Healthcare Australia Pathology) for testing. Genomic DNA is isolated, and pharmacogenomic genotyping is performed using either the iPLEX® PGx74 or VeriDose® Core panel (Agena Biosciences, San Diego, USA), which includes eight *CYP2C19* alleles (*2, *3, *4, *5, *6, *7, *8, *17) and 18 *CYP2D6* alleles (*2, *3, *4, *6, *7,*8, *9, *10, *11, *12, *14, *15, *17, *18, *19, *29, *41, *114). In addition, an in-house digital droplet PCR copy number assay is conducted to detect *CYP2D6* gene deletions (*5) and duplications (*XN). Genotype to metaboliser phenotype translation is performed according to the CPIC guidelines [12, 13] by the Translational Software.

#### Incorporation of interaction with concomitant medications into PGx phenotype

*CYP2D6* and *CYP2C19* metaboliser phenotypes are adjusted for participant-reported concomitant medications known to induce or inhibit these enzymes using the Sequence2Script tool [21]. In the presence of an inducer, the genotype-predicted phenotype is converted to the next higher activity phenotype (e.g. an intermediate metaboliser is converted to a normal metaboliser). In the presence of a moderate inhibitor, the genotype-predicted phenotype is converted to the next lower activity phenotype (e.g. a normal metaboliser is converted to an intermediate metaboliser), whereas in the presence of a strong inhibitor, the phenotype is converted to a poor metaboliser, regardless of the genotype-predicted phenotype.

#### Determination of algorithm of actionable recommendations 

Table 1 shows the algorithm for drug selection based on concomitant medication-adjusted PGx phenotype. The algorithm of recommendations includes contraindicated drugs and those where a dose alteration is recommended and was based on CPIC and DPWG antidepressant guidelines [11–13].

#### Selection of medications for inclusion in the antidepressant prescribing report 

The selection of medications and dosages to be included in the report is generated in R [22] using the algorithm in Table 1. Firstly, the number of medications selected for the report (between four and six) is randomly determined using the base R function *sample.* Variation in the number of drugs included in the report (four to six) was chosen to maintain blinding and allow the potential to include all genotype-based actionable recommendations in an intervention report. Drugs which are contraindicated according to the participant’s phenotype (‘not recommended’) are never included in the report. Medications with actionable recommendations (bold texted cells) are prioritised in the report, i.e. they are always included and listed at the top of the report. If there are more medications with actionable recommendations than the number of medications to be included in the report, then a random selection is taken. To fill the remaining medications in the report, a random selection of medications with no recommendations (non-bold texted cells) is taken.

#### Return of report to GPs

Reports are returned to the GP clinic via hard copy or secure file transfer. The GP clinic staff are asked to treat the report as per their standard procedures for receiving and actioning pathology test reports. GP clinic staff are asked to upload the report to the participant’s GP medical record.

Ultimately, any clinical decisions regarding the pharmacological or non-pharmacological treatment of depressive symptoms are at the discretion of the participants and their GP. This means that GPs are asked to employ their clinical decision-making as per usual clinical practice but are equipped with the antidepressant prescribing report to consider antidepressant treatment options. Responsibility for all aspects of participant care is the GP’s, as per standard of care.

**Table 1:** Algorithm for drug selection based upon concomitant medication-adjusted PGx phenotype in the preside

CYP2C19 phenotype	CYP2D6 phenotype	**Citalopram [SSRI] ** ***CYP2C19***	**Desvenlafaxine [SNRI]**	**Duloxetine [SNRI]**	**Escitalopram [SSRI] ** ***CYP2C19***	**Fluoxetine [SSRI]**	**Fluvoxamine [SSRI] ** ***CYP2D6***	**Mirtazapine [others]**	**Paroxetine [SSRI] ** ***CYP2D6***	**Sertraline [SSRI] ** ***CYP2C19***	**Venlafaxine [SNRI] ** ***CYP2D6***
Intermediate, normal or null	Intermediate	20 mg mane	50 mg mane	60 mg mane	10 mg mane	20 mg mane	50 mg nocte	15 mg nocte increase to	20 mg mane	50 mg mane	Not recommended
Poor	Intermediate	**10 mg mane**	50 mg mane	60 mg mane	**5 mg mane**	20 mg mane	50 mg nocte	30 mg nocte	20 mg mane	**25 mg mane**	Not recommended
Rapid or ultrarapid	Intermediate	Not recommended	50 mg mane	60 mg mane	Not recommended	20 mg mane	50 mg nocte	15 mg nocte increase to	20 mg mane	50 mg mane	Not recommended
Intermediate, normal or null	Normal	20 mg mane	50 mg mane	60 mg mane	10 mg mane	20 mg mane	50 mg nocte	30 mg nocte	20 mg mane	50 mg mane	75 mg mane
Poor	Normal	**10 mg mane**	50 mg mane	60 mg mane	**5 mg mane**	20 mg mane	50 mg nocte	15 mg nocte increase to	20 mg mane	**25 mg mane**	75 mg mane
Rapid or ultrarapid	Normal	Not recommended	50 mg mane	60 mg mane	Not recommended	20 mg mane	50 mg nocte	30 mg nocte	20 mg mane	50 mg mane	75 mg mane
Intermediate, normal or null	Null	20 mg mane	50 mg mane	60 mg mane	10 mg mane	20 mg mane	50 mg nocte	15 mg nocte increase to	20 mg mane	50 mg mane	75 mg mane
Poor	Null	**10 mg mane**	50 mg mane	60 mg mane	**5 mg mane**	20 mg mane	50 mg nocte	30 mg nocte	20 mg mane	**25 mg mane**	75 mg mane
Rapid or ultrarapid	Null	Not recommended	50 mg mane	60 mg mane	Not recommended	20 mg mane	50 mg nocte	15 mg nocte increase to	20 mg mane	50 mg mane	75 mg mane
Intermediate, normal or null	Poor	20 mg mane	50 mg mane	60 mg mane	10 mg mane	20 mg mane	**25 mg nocte**	30 mg nocte	**10 mg mane**	50 mg mane	Not recommended
Poor	Poor	**10 mg mane**	50 mg mane	60 mg mane	**5 mg mane**	20 mg mane	**25 mg nocte**	15 mg nocte increase to	**10 mg mane**	**25 mg mane**	Not recommended
Rapid or ultrarapid	Poor	Not recommended	50 mg mane	60 mg mane	Not recommended	20 mg mane	**25 mg nocte**	30 mg nocte	**10 mg mane**	50 mg mane	Not recommended
Intermediate, normal or null	Ultrarapid	20 mg mane	50 mg mane	60 mg mane	10 mg mane	20 mg mane	50 mg nocte	15 mg nocte increase to	Not recommended	50 mg mane	**75 mg CR mane increase to 225 mg**
Poor	Ultrarapid	**10 mg mane**	50 mg mane	60 mg mane	**5 mg mane**	20 mg mane	50 mg nocte	30 mg nocte	Not recommended	**25 mg mane**	**75 mg CR mane increase to 225 mg**
Rapid or ultrarapid	Ultrarapid	Not recommended	50 mg mane	60 mg mane	Not recommended	20 mg mane	50 mg nocte	15 mg nocte increase to	Not recommended	50 mg mane	**75 mg CR mane increase to 225 mg**

### Criteria for discontinuing or modifying allocated interventions {11b}

The trial intervention only contains the provision of the prescribing report to the GP clinic, and therefore, no substantive modifications are anticipated. Participants, in collaboration with their GP, are free to take up their treatment recommendation or not and may discontinue treatment at any time.

### Strategies to improve adherence to interventions {11c}

Upon recruitment, participants are informed that the prescribing report will be provided to their GP after 2–3 weeks and that they should make an appointment with their GP at this time to discuss its recommendation and the management of their depressive symptoms. In the event of any substantial delay to the PGx results and therefore the antidepressant prescribing report, participants and GPs are informed of this delay and when to expect the report.

### Relevant concomitant care permitted or prohibited during the trial {11d}

Those who are taking antipsychotic medication at baseline are ineligible for the trial, as described above; however, those who begin treatment with antipsychotic medication during their study participation are not excluded. There are no other exclusions based on concomitant care.

### Provisions for post-trial care {30}

There are no anticipated harms associated with the intervention, given that the participant and their GP have final responsibility for any clinical decisions and care and all antidepressant medication recommendations are taken from the Australian Therapeutic Guidelines. GPs are under no obligation to use the recommendations on the provided prescribing report. All participants are invited to discuss their participation with their GP and any mental health symptoms they may be experiencing. Therefore, there are no provisions for post-trial care.

### Outcomes {12}

Outcome measures are collected at baseline prior to randomisation and then at 4, 8, 12 and 26 weeks after the GP’s receipt of the antidepressant prescribing report. Further details about measures can be found in item 18a (Plans for assessment and collection of outcomes) below.

#### Primary outcome measures

Difference between the experimental and control interventions in the mean change of depressive symptom score from baseline to 12 weeks from the GP’s receipt of the antidepressant prescribing report. The depressive symptoms score is the sum of the nine items measured using the Patient Health Questionnaire 9 (PHQ-9) [19].

#### Secondary outcomes measures

Difference between the experimental and control interventions in the:

(i) mean change in PHQ-9 depressive symptom scores from baseline to 4, 8 and 26 weeks from the GP’s receipt of the antidepressant prescribing report
(ii) Proportion of participants in remission from depressive symptoms (defined as PHQ-9 score < 5) at 12 weeks
(iii) Proportion of participants who respond to treatment, defined as > 50% decrease in PHQ-9 score from baseline, at 12 weeks
(iv) The mean side effect score due to antidepressant medications at 4, 8, 12 and 26 weeks, measured using the FIBSER scale [23], that includes the domains of frequency, intensity and burden of side effects
(v) Quality of life, measured as the mean AQoL-4D [24] utility score, at 12 and 26 weeks (exploratory analyses of the sub-domains of his scale, including the mental health dimension, will also be undertaken)
(vi) The mean self-reported adherence score to antidepressants prescribed, measured using the MARS-5 scale [25], at 4, 8, 12 and 26 weeks
(vii) Adherence to antidepressants prescribed, measured using the medication possession ratio [26], derived from prescription and PBS data
(viii)  Number of antidepressant medication changes, derived from GP record audit and PBS data
(ix) Proportion of participants where the GP prescribing was concordant with the medication recommendations in the antidepressant prescribing report

##### Economic evaluation

Health economic outcomes will be measured as the difference between the experimental and control interventions in the following:

(i) Quality-adjusted life years (QALYs) calculated using AQoL-4D utility values and the area under the curve method
(ii) Health service use, measured using a fit-for-purpose resource use questionnaire [27, 28], MBS and PBS data, and GP record audit, at 12 and 26 weeks
(iii) Lost productivity from paid and unpaid work and presenteeism (time working but at a reduced capacity) measured with questions in the resource use questionnaire
(iv) Total health sector costs calculated by adding the cost of intervention delivery to participant health care service use
(v) Total partial societal costs calculated by adding the total cost of lost productivity to total health sector costs

##### Process evaluation

A process evaluation, based on a logic model of how the intervention is designed to affect the outcome, will also be conducted to further explore how elements of the trial intervention influenced potential outcomes. This will be measured using qualitative data from semi-structured interviews from a subset of general practitioners and participants enrolled in the trial, as well as documented participant and GP interactions regarding mental health throughout the trial period, obtained from GP electronic medical record audit.

### Participant timeline {13}

Table 2 shows the participant timeline from the time of enrolment and the timing of the different assessments. Twenty-six weeks post-allocation (i.e. after the final endpoint of the study), all participants’ GPs receive a full clinical PGx report that outlines PGx-guided prescribing recommendations for a range of commonly prescribed medications.

**Table 2:** PRESIDE trial participant timeline

	**Trial period**						
	***Enrolment***	***Allocation to intervention***	***Post-allocation***				
Time point	0 weeks	2–3 weeks	4 weeks	8 weeks	12 weeks	26 weeks	Post-26 weeks
Enrolment	X						
* Eligibility screen*	X						
* Informed consent*	X						
* DNA collection*	X						
* Allocation to intervention*		X					
Interventions							
* Antidepressant prescribing report*		X					
* Full clinical PGx report*							X
Assessments							
* Demographics*	X						
* PHQ-9*	X		X	X	X	X	
* FISBER*	X		X	X	X	X	
* AQoL-4D*	X				X	X	
* MARS-5*	X		X	X	X	X	
* COVID-19 QoL and Mental Health Impact Scale*						X	
* Resource use questionnaire*	X				X	X	
* GP record audit*						X	
* MBS and PBS data*							X

### Sample size {14}

Sample size estimates were informed by two large randomised trials in which 1868 participants (Target-D [29]) and 1671 participants (Link-me [30]) with depressive symptoms attending general practice. We would require a sample size of 672 eligible patients to be randomised (336 patients in each experimental and control intervention) to detect a between-arm difference of 0.3 standard deviation (SD) for the primary outcome, with 90% power and a 5% significance level (2-sided test), after allowing for 30% attrition over 12 weeks. This is equivalent to a difference in the mean change of PHQ-9 score of 1.8 at 12 weeks (measured from baseline) between the two study interventions, assuming conservatively the standard deviation is 6 [29, 30]. A reduction of at least 0.3 SD in the mean PHQ-9 depressive symptom score at 12 weeks between study interventions is considered a clinically important reduction in the primary care setting.

From our previous trials and experience [29, 30], we expected that 40% of all patients approached would complete the PHQ-9, of whom 32% would be eligible due to moderate to severe depressive symptoms. Of these eligible patients, we expected 40% would consent to enter the trial. Therefore, we predicted 13,125 patients will need to be approached to reach the required sample size.

### Recruitment {15}

#### Identification of potential participants

Patients from appointment lists of consented GPs are sequentially approached. The approach occurs either via telephone up to two days prior to their scheduled GP appointment or in person in the waiting room immediately before their scheduled GP appointment.

##### Telephone approach

Potential participants are first approached via SMS text to notify in advance that a researcher based in their GP clinic will be calling them to discuss the study. They are then phoned to introduce the study and screen them for eligibility, including completing the PHQ-9 over the telephone. Research assistants attempt to call potential participants a maximum of two times. A voicemail may be left after the first attempt. If the potential participant is eligible and interested in hearing more about the study, they are then asked to attend their GP appointment 30 min early (in person or virtually for telehealth appointments) to meet with a second researcher to discuss further what the study involves. After the initial phone call, they are emailed a copy of the study information sheet.

##### Face-to-face approach

Potential participants are first approached in the waiting room immediately prior to their GP appointment. If they are willing, they are provided with a tablet to complete the PHQ-9 questionnaire and other eligibility questions. If they are eligible for the study, they are invited to a private consulting room with another researcher to confirm their eligibility and discuss the study further.

#### Participant recruitment and consent

Given the sensitive nature of the topic being discussed with participants, the recruitment appointment can only be scheduled on the day of the participant’s existing GP appointment, ideally immediately prior to the GP appointment. Recruitment can occur either face-to-face at the participant’s GP clinic or via teletrial.

##### Face-to-face recruitment and consent

Interested potential participants meet with the researcher before their scheduled GP appointment, in a private consulting room. After confirmation of eligibility, the trial is explained and the potential participant is given the opportunity to ask questions, followed by informed consent to participate. During this appointment, the participant signs the hard copy study consent form, as well as the optional Services Australia consent form (for access to Medical Benefits Scheme and Pharmaceutical Benefits Scheme data), provides a sample of DNA using the saliva collection kit, completes the baseline questionnaire and is provided with an alert card to give their GP to inform the GP they are part of the trial. Participants are informed they will be required to schedule a follow-up appointment after 2–3 weeks to discuss the antidepressant prescribing report with their GP.

##### Teletrial recruitment and consent

Interested potential participants who cannot attend their clinic (either for convenience reasons or due to COVID-19 lockdowns) can consent to the trial via teletrial, using online videoconferencing software. Confirmation of eligibility and obtaining informed consent are as per face-to-face protocols. The study consent form is completed as an e-consent form [31] via the study’s REDCap database, as is the baseline questionnaire.

After this initial consent appointment, the participant is express-posted a hard copy of the Services Australia consent form (which cannot be completed electronically) and the DNA saliva collection kit. Once received by the participant, a researcher has another videoconferencing appointment with the participant to witness them complete the DNA collection (to ensure its correct identity and sample integrity). The Services Australia consent form and DNA sample are then express-posted back to the research team for processing and logging.

#### Ineligible patients and patients who do not wish to participate in the trial

An electronic recruitment log containing age and gender is kept throughout recruitment. Reasons for ineligibility or refusal (if provided) are recorded in REDCap. No identifying data is kept for this group. This recruitment log is maintained to track the representativeness of the trial sample.

## Assignment of interventions: allocation

### Sequence generation {16a}

Participants are randomly allocated 1:1 to the experimental and control intervention. The allocation sequence is computer-generated, stratified by general practice and current antidepressant use using permuted blocks of random sizes. To ensure concealment, the block sizes are not disclosed until after recruitment of trial participants is completed.

### Concealment mechanism {16b}

The random allocation schedule is embedded within a secure online web database (REDCap [20]) which automatically randomises participants to either experimental or control intervention after their DNA results have been returned to the investigator team.

### Implementation {16c}

A statistician not involved in the recruitment of participants or data collection generates the randomisation schedule and upload it to the trial online database. Researchers randomise participants upon receipt of their PGx test result, immediately prior to generating the antidepressant prescribing report, using the randomisation function in the REDCap database [20]. Allocated intervention is hidden from this researcher using the Hide Randomisation Module v1.0.4 in REDCap.

#### Protocol modification

Until 26 August 2021 and the 65th randomised participant, randomisation occurred upon receipt of the study consent form. However, seven of the initial PGx test samples failed genotyping in the laboratory, which then resulted in the withdrawal of four of these participants who did not wish to provide a blood sample for repeat testing. At this point, the decision was made by the trial steering committee on 26 August 2021 to randomise participants only on receipt of complete PGx results, due to concerns of a large number of patients not able to receive the antidepressant prescribing report at all that may lead to an attenuation in the intervention effect. Although there are some pragmatic elements in the design of this trial, we wanted to maximise the chance of demonstrating an effect of the experimental intervention compared to the control intervention. For this aspect of the trial, the design is more explanatory [32]. Any participants who were recruited and randomised prior to this will all be included in the final analysis, under the intention-to-treat principle, regardless of if they received the full intervention.

## Assignment of interventions: blinding

### Who will be blinded {17a}

Only researchers who randomise participants and generate the antidepressant prescribing reports are unblinded to participants allocated intervention. All others involved in the trial are masked to participants’ study intervention allocation. This includes trial participants, GPs (who act upon the antidepressant prescribing report), other researchers who recruit and follow-up participant questionnaires, all other researchers in the trial steering committee overseeing the conduct and running of the trial and the trial statistician. The trial online database (REDCap [20]) restricts access to participants’ study intervention allocation for all researchers who do require it, using the user rights options. Masking at the time of trial results, analyses will be maintained by randomly designating an uninformative code to each of the study interventions. The results of the trial will initially be presented to the trial steering committee using the uninformative code to maintain masking and will be revealed after the results have been interpreted.

### Procedure for unblinding if needed {17b}

#### On trial 

While on the trial, there will be no unblinding, given the clinical care of participants is always at the discretion of GPs and any antidepressant recommendations after within the Australian Therapeutic Guidelines.

#### On completion of the trial 

Explicit unblinding of participants will not occur at the completion of the trial. However, the full clinical PGx report is sent directly to GPs at the completion of the participant’s involvement in the trial. Review and discussion of these reports is the responsibility of the GP, as per a regular pathology report. At this stage, it may be possible to determine the participant’s study intervention by looking for discrepancies between the trial and more extensive clinical report. Unblinding of researchers and investigators not involved in the participant recruitment, including the statistician responsible for the analyses, will occur after the primary statistical analyses have been completed and results interpreted.

## Data collection and management

### Plans for assessment and collection of outcomes {18a}

#### Questionnaire measures 

Questionnaire data from participants is collected using a dedicated REDCap database [20]. At baseline, this is completed by the researcher in person, or via a teletrial video call. Participants are then asked for their preference to complete subsequent questionnaires (4, 8, 12 and 26 weeks) via email (sent on the due date, with a link to the REDCap survey), via a post on hardcopy (sent 5 days prior to the due date) or via phone (where the researcher calls the participant on the due date and enters data directly into the REDCap survey).

##### Demographics

Participants’ demographics are collected directly from participants at baseline, including gender, age, language mainly spoken at home, ethnicity, highest level of education, employment status and living arrangements. Additionally, smoking status, alcohol intake and cannabis use are collected. Categories provided for these questions are derived from the Australian census [33].

##### Medication use

Current medications (prescribed and over-the-counter) are collected via self-report from participants at baseline and current antidepressant use (yes/no) at all questionnaire time points.

##### PHQ-9

The Patient Health Questionnaire 9 (PHQ-9) measures the severity of depressive symptoms [19]. The PHQ-9 assesses the nine symptoms of depression, outlined in the Diagnostic and Statistical Manual of Mental Disorders, over the last 2 weeks using a 4-point Likert scale. Total scores calculated by adding the 9 items range between 0 and 27 with cut-points of 5, 10, 15 and 20 indicating mild, moderate, moderately severe and severe depressive symptoms, respectively. The PHQ-9 is a validated, self-reported diagnostic measure in primary care [34] with demonstrated efficacy and sensitivity as an outcome measure for treatment trials with a recommended Reliable Change Index [35].

##### FIBSER

The FIBSER (Frequency, Intensity, Burden of Side Effects Rating) is a 3-item validated self-reported symptom checklist [23]. Participants rate how often they experience side effects they attribute to their medication and how severe these side effects are and the degree to which they interfere with daily functioning. Each domain (frequency, intensity and burden) is rated on a 7-point scale and assessed separately.

##### MARS-5

The Medication Adherence Report Scale (MARS-5) measures patient adherence to antidepressant prescribing [25]. It is a 5-item self-reported scale, with each item indicating elements of non-adherence rated as never (5), rarely (4), sometimes (3), often (2) and always (1). Scores are summed to give a total score ranging between 5 and 25, with higher scores indicating higher levels of reported adherence. The MARS-5 is a validated scale and has been shown to have good reliability and validity across health conditions [25].

##### AQoL-4D

The Assessment of Quality of Life 4 Dimension (AQoL-4D) is a 12-item scale that measures four domains of health-related quality of life: independent living, mental health, relationships and senses [24]. The AQoL-4D is validated and is scored using a preference-weighted scoring algorithm to derive a utility score between 0 and 1 used to calculate quality-adjusted life years (QALYs) for cost-effectiveness analyses.

##### COVID-19 impact scale

The PRESIDE Trial commenced during the COVID-19 pandemic (first participant recruited in May 2021) and some of the participant recruitment occurred during strict lockdown restrictions in Victoria, Australia. The impact of the pandemic and associated public health interventions on the mental health of the population is now well documented [36]. Given some participants were recruited during lockdowns and some when public movement and social interaction were restricted, we hypothesised that this could potentially impact the proportion of potential participants who were eligible for the study (i.e. scored ≥ 10 on the PHQ-9 scale), or the nature of depressive symptoms in those eligible (i.e. situational-based depression versus long-standing, potentially refractory depression). It could also affect GP and patient decisions to take antidepressants or use psychological therapies instead.

Therefore, on 25 November 2021, it was proposed that an additional measure should be collected to determine the perceived impact of the pandemic and lockdowns on the mental health of trial participants and the effect it may have had on their depressive symptoms and their treatment. The COV19 – Impact on Quality of Life [37] measure was selected as it asks participants to reflect on the impact COVID-19 has had on their mental health. It is a six-item scale that has been validated in a European general population and clinical sample. Each item is answered on a scale of strongly disagree (1) to strongly agree (5) regarding the impact that the spread of coronavirus has had on aspects of participants’ mental health. Scores are averaged to determine a total score. A higher score indicates a greater perceived impact of the pandemic on one’s quality of life. This measure was added to the 26-week questionnaire, as at the time of making this decision (25 November 2021), no participants had reached this time point, facilitating the collection of these data for the entire study cohort.

##### Resource use questionnaire

A fit-for-purpose resource use questionnaire, used in our previous studies [27, 28], has been included in the study measures. This questionnaire covers access to medical and mental health professionals; self-help measures, such as the use of mobile apps or internet support; and impact of mental health symptoms on paid and unpaid work and presenteeism.

### Administrative health service use data collection

#### Medicare Benefits Schedule (MBS) and Pharmaceutical Benefits Scheme (PBS) data collection 

MBS and PBS data are administrative datasets that contain information on services and medications that qualify for a benefit under the Australian Health Insurance Act and for which a claim has been processed. These datasets for study participants will be requested from Services Australia for all participants providing consent for the period of 1 year prior to and 1 year after their consent date. This data includes services provided by doctors and allied health professionals (i.e. general practitioners, psychiatrists, psychologists), diagnostic tests (i.e. pathology and imaging) and prescription medications dispensed.

#### GP record audit 

Participants’ GP records are audited by researchers blinded to the trial allocation of the participants for all consultations where their mental health was discussed from the date of consent to 26 weeks after the GP’s receipt of the antidepressant prescribing report (the final endpoint of the study). The audit collects the date of consultations; any mental health diagnoses; discussion of the antidepressant prescribing report; discussion of the use of antidepressants, including current antidepressants and their effect on symptoms and side effects; discussions of commencement or change of antidepressants; and final prescription of antidepressants. These data are entered by researchers into the study REDCap database while at general practices.

#### Process evaluation 

The process evaluation, based on the logic model of the proposed effect of the intervention on the outcome, aims to explore the barriers and facilitators of pharmacogenomic testing for antidepressant use in primary care. This evaluation will also explore the underlying assumptions of the proposed logic model. It will be conducted using data on consultations between the participant and their GP regarding their mental health in the study follow-up period, as well as semi-structured interviews for qualitative responses from general practitioners and participants enrolled in the study through individual interviews. The interviews will be conducted with 15–20 GPs and 15–20 participants.

A purposive sample of participants is being recruited according to age, gender, time point in the trial and whether they were newly prescribed an antidepressant within the trial or had their treatment altered. Participants do not need to have finished their trial period to participate as they are blinded to trial group allocation.

We are also interviewing a purposive sample of GPs whose patients are in the trial, to explore their perspectives on the use of pharmacogenomic testing to inform their antidepressant prescribing. This covers their understanding of the test, preferences for reporting and recommendations, their use of the trial prescribing reports and impact on their prescribing decisions, potential impact on the therapeutic alliance with their patients and future models of implementation into routine practice. GPs must not have active participants in their trial period to participate, all the pharmacogenomic test results must have been obtained by the GP for their patients.

Consent to be contacted for these interviews is indicated in the study consent form and additional recorded verbal consent for the interview is recorded. Interviews are undertaken either in person or via videoconferencing software. All interviews are transcribed by an automated programme with researcher review, or by a professional transcription service.

Interviews are informed by a topic guide (i.e. interview schedule) based on relevant literature and revised based on emerging findings from the iterative analytic process. Interviews are audio recorded (if videoconferencing software is used, video is used during the interviews so that the interviewer can respond to non-verbal cues, but video recordings are not stored).

Interviews will be analysed using thematic analysis. Themes arising from the interviews will be organised and coded using a qualitative data analysis software (e.g. NVivo [38]). At least two researchers will be involved in the coding and analysis.

### Plans to promote participant retention and complete follow-up {18b}

During the baseline appointment, participants are asked for a preferred format for their follow-up questionnaire (email/post/telephone). In the case of participants who do not complete the follow-up questionnaire at 4, 8, 12 and 26 weeks, a further three attempts are made to contact them via phone, email or SMS. If no response is obtained within 2 weeks (for the 4- and 8-week questionnaires) or 5 weeks (for the 12- and 26-week questionnaires), the participant is considered a non-responder for that questionnaire. Participants that do not respond to a specific questionnaire are still invited to complete their subsequent follow-up questionnaires, unless they explicitly have withdrawn from the trial.

### GP and participant withdrawal

Participants and GPs can withdraw from the study at any time without giving a reason, as stated to them prior to informed consent and within the consent form.

Participants can withdraw from further contact from the trial team (i.e. from questionnaire follow-up). They also have the option to withdraw any unprocessed data at the time of withdrawal. If a participant withdraws prior to the saliva sample being analysed, their DNA sample is destroyed and they do not receive the full PGx report at the conclusion of their 26 weeks of participation. Participants who withdraw after their sample has been analysed and are randomised have their full PGx report sent to their GP, unless they opt not to, or they opt to remove all their unprocessed data. If participants withdraw from contact only, their objective health service use data is still collected as this does not require contact with the participant. If participants opt to withdraw their data, all their unprocessed data is destroyed.

### Data management {19}

Data are collected, managed and stored according to the study’s data management plan, developed in accordance with the University of Melbourne’s (UoM) Research Data Management Policy and Research Code of Conduct. A REDCap online database is used to collect and store data, only accessible by authorised and trained researchers. REDCap is a password-protected online database that has mandatory data entry fields to reduce missing data, range checks for the data values and branching questions [20]. Before randomisation, REDCap provides a pop-up for researchers to double-check data entry of the variables used for stratifying randomisation. All paper-based data is entered directly into REDCap by researchers blinded to arm allocation and these are stored securely in an office within UoM offices, under the responsibility of the study principal investigator (JE) in a locked file cabinet. All data is only accessible to researchers listed on ethical approvals.

### Confidentiality {27}

Prior to consent, any identifiable information about potential participants does not leave their general practice and is not retained by researchers.

Participant confidentiality is strictly held in trust by the principal and study investigators, research staff and the sponsoring institution and their agents. This confidentiality is extended to cover testing of biological samples and genetic tests in addition to the clinical information relating to participants.

To preserve confidentiality and reduce the risk of identification during the collection, analyses and storage of data, the following are undertaken:

- Minimal sensitive and health information is collected on participants. The data collected is limited to that required to address the primary and secondary objectives.
- Participant identifiers are stored securely with restricted access using REDCap’s permission control functionality. Where possible, participant data is identified through the use of a unique participant study ID assigned to the participant (“re-identifiable”). The study coordinator is responsible for the management of REDCap’s permission control functionality and restricting access to participant identifiers to those who a directly involved in participant follow-up.
- The trial statistician conducting the analyses will be provided with anonymised data using a unique participant trial ID.
- All DNA sample specimens and associated forms are transported to the testing laboratory through Melbourne Pathology (Sonic Healthcare), using a courier. Upon receipt by Melbourne Pathology, a unique identifier (episode ID) is allocated to each sample. This episode ID, along with the participant trial ID, then accompanies all data through the genotyping and phenotyping process, including return to researchers at UoM. These two unique identifiers then allow for reidentification of the data by UoM researchers, without the need to send personal identifiers.
- All data is managed according to UoM’s Research Data Management Policy and Research Code of Conduct, including security protocols such as two-factor authentication and storage on secure servers.

This research involves the linkage of data sets with the consent of participants. Participants are advised that identifying data is collected and provided to respective government agencies and departments to facilitate linkage. Participants provide separate written informed consent for the team to access MBS and PBS data. The extent to which identifying information is shared to each agency and department is outlined in the consent process.

### Plans for collection, laboratory evaluation and storage of biological specimens for genetic or molecular analysis in this trial/future use {33}

DNA is collected with ORAcollect®-DNA OCR100 saliva collection kits (DNA Genotek, Ottawa, ON, Canada). DNA samples are logged on the REDCap database by the UoM team and then sent to Melbourne Pathology (Sonic Healthcare Australia Pathology) by courier. Sample management at Sonic Healthcare is according to their standard approved protocols, given the clinical nature of the sample and test (NATA accredited). DNA samples are disposed of by Sonic Healthcare’s standard operating procedures. Samples are not returned to UoM for storage.

If the original DNA saliva sample does not yield enough quantity or quality of DNA, then another DNA sample is required. This second sample is a blood sample, given the much smaller chance of insufficient DNA.

## Statistical methods

### Statistical methods for primary and secondary outcomes {20a}

Descriptive statistics will be used to summarise the baseline characteristics of participants by the experimental and control interventions. Primary analyses will include all randomised participants using an intention-to-treat principle, where they will be analysed in the study intervention that they were assigned, regardless of whether they received all, part, or none of the intended intervention.

For the primary outcome, a linear mixed-effects model using restricted maximum likelihood with random intercepts for individuals will be used to estimate the mean difference between experimental and control interventions in the mean change of depressive symptoms from baseline at each follow-up time point. The model will adjust for general practice, antidepressant use at baseline, ancestry (if imbalanced as *CYP2C19* and *CYP2D6*, phenotype frequency varies by ancestry) and time (4, 8, 12 and 26 weeks), with a two-way interaction between study intervention and time. The model will also adjust for baseline depressive symptoms which will be constrained to be equal between the two study interventions. Estimates of the intervention effect will be reported as the mean difference between the experimental and control interventions, with 95% confidence intervals and *p*-value. There will be no adjustment for handling the multiplicity of testing and control for the final type I error rate.

The same approach will be undertaken using a linear mixed-effects model between experimental and control interventions for continuous secondary endpoints. Similar regression analyses appropriate to the data type (e.g. logistic for binary, Poisson for count data) will be performed on other secondary endpoints. Analyses for the secondary endpoints will be described in detail in a statistical analysis plan (SAP), which will be made available on the trial registry prior to the primary analysis. Analyses will be conducted in Stata 17.0 [39] and R [22].

#### Economic evaluation

The economic evaluation will be undertaken from the health sector and partial societal perspectives. The health sector perspective includes costs borne by the government as a third-party payer in addition to out-of-pocket costs incurred by patients when accessing health care. This includes the estimated cost to deliver the PGx-informed antidepressant prescribing combined with the cost of additional health services used by participants over the time period of the trial. The partial societal perspective adds the cost of lost productivity (absenteeism and presenteeism) for study participants to health sector costs. Incremental cost-effectiveness ratios (ICERs) will be calculated as the difference in the average total cost between the randomised arms, divided by the difference in the average outcome. The outcomes used in these analyses will include the primary outcome of the PHQ-9 score and QALYs calculated from AQoL-4D utility values using the area under the curve method. ICERs using other secondary study outcomes (e.g. cost per remitted case) will also be explored. Confidence intervals around ICERs will be calculated using a nonparametric bootstrap procedure, with 1000 iterations to reflect sampling uncertainty. The bootstrapped ICERs and the CIs will be graphically represented on cost-effectiveness planes. A cost-effectiveness plane is a plot of the 1000 bootstrapped incremental costs and outcomes across four quadrants. Acceptability curves will be used to graphically present the proportion of bootstrapped iterations falling below a specific willingness to pay threshold. The Productivity Commissions range of willingness to pay thresholds will be used to assess cost-effectiveness [40]. Ratios under $33,000/QALY are deemed very cost-effective, between $33,000 and $64,000 per QALY gained cost-effective and between $64,000 and $96,000 per QALY gained marginally cost. Ratios greater than $96,000 per QALY gained are not considered cost-effective.

Sensitivity analyses will be used to determine the impact of changes to important study parameters (e.g. unit cost price variation including the cost of genotyping in this trial).

A modelled budget impact analysis using the results of this trial will be undertaken to estimate the costs of implementing the PGx-informed antidepressant prescribing at a state or national level.

### Interim analyses {21b}

No interim analyses are planned.

### Methods for additional analyses (e.g. subgroup analyses) {20b}

Sensitivity analyses will also test the robustness of the result to variations in the underlying assumptions and inputs to the health economic analysis. Further supplementary analyses, including sensitivity analyses and pre-planned sub-group analyses, will be described in the SAP.

### Methods in analysis to handle protocol non-adherence and any statistical methods to handle missing data {20c}

Details of the compliance-adjusted analysis and appropriate methods for dealing with missing endpoint data will also be provided in the SAP.

### Plans to give access to the full protocol, participant-level data and statistical code {31cI}

To assist with reproducible research, the full protocol, non-identifiable participant-level data and statistical code will be made available to external researchers upon reasonable request. The trial steering committee will manage external requests for these materials.

## Oversight and monitoring

### Composition of the coordinating centre and trial steering committee {5d}

All meetings, including of the trial steering committee (SS, PC, CM, MLC, JG, TC, TP, ED, MG, CD, JE) and trial management (SS, PC, CM, MLC, TC, TP, ED, LH, NM, TS, MG, CB, JE) group, will be organised, recorded (as appropriate) and minuted by the coordinating centre. ED is a lived-experience researcher and brings the stakeholder and public perspective to the trial steering committee and trial management group.

#### Coordinating centre 

The coordinating centre primarily comprises the research team who liaises with general practice clinics and oversees the day-to-day management of the trial (SS, AA, RB, LS, PA, GR, ZS, JL, RS, PL and JE). The research team is supervised by the trial coordinator (SS), and the overall responsibility and decision-making is with the chief investigator (JE). The research team, including the trial coordinator and chief investigator, is responsible for implementing and executing the trial including general practice recruitment, patient recruitment, governance and administration, data collection, management of adverse events and document management.

#### Trial management group 

The chief investigator is responsible for supervising any individual or party to whom they have delegated tasks for the trial. Delegated tasks and roles will be recorded on a delegation log. They provide continuous supervision and documentation of their oversight. To meet this GCP requirement, a small group will be responsible for the day-to-day management of the trial, led by the trial coordinator who will delegate and provide daily supervision to the research team. The research team at the coordinating centre meet 4–6 weekly with external researchers and laboratory staff (CB, MG, Sonic Healthcare, Translational Software) for oversight of the day-to-day trial. The group closely reviews all aspects of the conduct and progress of the trial, ensuring that there is a forum for identifying and addressing issues. Meetings are minuted with attendees listed, pertinent emails retained, and phone calls documented.

#### Trial steering committee 

A trial steering committee has been established to provide expert advice and overall supervision and ensure that the trial is conducted to the required standards. The steering committee includes the chief investigators, associate investigators and the research team. The steering committee meets quarterly, with more frequent meetings added as required throughout the duration of the trial set-up, recruitment and post-recruitment analysis phase. All meetings are minuted and digitally stored with all trial documentation.

### Composition of the data monitoring committee, its role and reporting structure {21a}

We do not expect significant adverse effects arising from the trial itself, as clinical management of all participants is the responsibility of their GP and treating team. We have therefore decided not to have a separate data monitoring committee. Oversight of the trial will be managed by the trial steering committee.

### Adverse event reporting and harms {22}

All protocol deviations are recorded in the participant record and reported to the study coordinator and lead investigator (SS and JE), who will assess for seriousness. Those deviations deemed to affect to a significant degree the rights of a trial participant or the reliability and robustness of the data generated in the clinical trial are reported as serious breaches. Reporting is done in a timely manner (within 72 h to the study coordinator and lead investigator) and within 7 days to the site’s Research Governance Office. The study coordinator and lead investigator must review and report serious breaches to the approving Human Research Ethics Committee (HREC) within 7 days. Where non-compliance significantly affects participant protection or the reliability of results, a root cause analysis will be undertaken, and a corrective and preventative action plan prepared. Where protocol deviations or serious breaches identify protocol-related issues, the protocol is reviewed and, where indicated, amended.

### Frequency and plans for auditing trial conduct {23}

Researchers in the coordinating centre meet at least weekly with the chief investigator to discuss and review the trial progress. The chief investigator is contactable for prompt reporting of adverse events. The steering committee meets quarterly, with more frequent meetings added as required throughout the duration of the trial set-up, recruitment and post-recruitment analysis phase. Minutes of all meetings are digitally stored with all trial documentation. Progress is reported to the trial funder every 12 months. There is no independent auditing of trial conduct.

### Plans for communicating important protocol amendments to relevant parties (e.g. trial participants, ethical committees) {25}

This trial is conducted in compliance with the current version of the protocol. Any change to the protocol document or informed consent form that affects the scientific intent, trial design, participant safety, or may affect a participant’s willingness to continue participation in the trial is considered an amendment and therefore is written and filed as an amendment to this protocol and/or informed consent form. All such amendments are submitted to the HREC for approval prior to being implemented.

### Dissemination plans {31a}

Data from this trial will be disseminated in several ways. Informal dissemination of results will occur with participants, participating GPs and other collaborators. Participants in the study are given the option at the time of consent to receive a plain language, one-page summary of the study findings after statistical analyses are completed. Other collaborators will receive a similar summary, tailored to their position and interests (i.e. consumers will receive a lay summary).

The results of this research will be published in peer-reviewed journals. Upon publication of the results of the trial, we will generate media releases to health professionals and general outlets, generate Twitter and other social media content, and engage with health professionals and general podcasts. We will use all these approaches to promote the trial results. The chief investigators of the study hold primary responsibility for the publications of the results of the trial.

## Discussion

The PRESIDE Trial aims to determine if personalised antidepressant prescribing in primary care based on pharmacogenomic testing decreases depressive symptoms and increases remission from depression, reduces side effects and therefore improves adherence to antidepressants. Given that the vast majority of antidepressant prescribing occurs in primary care, evidence of the clinical utility of pharmacogenomics for antidepressants from tertiary psychiatric settings may not be sufficient to justify its routine use in general practice. Additionally, this trial will provide data on longer-term effects and impact on health service use, providing evidence on potential cost-effectiveness.

This trial began recruitment during the COVID-19 pandemic, and the recruitment period has included long periods of stay-at-home restrictions. This meant that both researchers and potential participants were subject to movement restrictions. The teletrial recruitment methods described above were employed, in part, to ensure recruitment to the study could continue during these lockdowns. These teletrial methods were designed to ensure that teletrial recruitment mirrored face-to-face recruitment as closely as possible, including witnessing via videoconferencing software the self-collection of the DNA samples.

The PRESIDE Trial was initially designed and funded prior to the emergence of the COVID-19 pandemic. As well as the global burden on mortality and morbidity from the disease itself, we now know that it has had a substantial impact on the mental health of many [36]. We do not know how this impact will affect the results of this trial, over and above the effects of the intervention. Upon discussion with the trial steering committee, we included the impact of the COVID-19 scale partway through the study, which will allow for exploration of whether there is an effect modification of the intervention between those whose depressive symptoms may be a result of the pandemic and those with other aetiology. It is difficult to hypothesise whether this may be the case and if so, in what direction this effect may go, as we do not yet understand whether antidepressant prescribing patterns may be different in this group (i.e. whether GPs may have been more or less likely to prescribe antidepressants to those experiencing depressive symptoms due to the social isolation or anxiety resulting from the pandemic) and the general efficacy of antidepressants on symptoms in this group. Regardless, the collection of this specific impact questionnaire will allow for the exploration of these hypotheses.

The collection of secondary quantitative outcomes using questionnaires, as well as the process evaluation including in-depth qualitative interviews, will allow for a thorough examination of the elements effect of this complex intervention. The development of an intervention logic model maps the potential points of the effect of the intervention and this mixed-method collection of process data will assist in the interpretation of the results of the trial.

This trial will provide evidence as to whether PGx-informed antidepressant prescribing is clinically efficacious and cost-effective. It will inform national and international policy and guidelines about the use of PGx to select antidepressants for people with moderate to severe depressive symptoms presenting in primary care.

## Trial status

Protocol Version 1.2, July 2022. The first participant was recruited on 26 May 2021. Trial recruitment is estimated to be completed in July 2023.

## Data Availability

SS and PC will have access to the final trial dataset. Non-identifiable data may be provided on request of external researchers after publishing the findings. The trial steering committee will manage external requests for data.

## Abbreviations

ANZCTR: Australian and New Zealand Clinical Trials Registration
AQoL: Assessment of Quality of Life
ATG: Australian Therapeutic Guidelines
COVID-19: Coronavirus disease 2019
CPIC: Clinical Pharmacogenetics Implementation Consortium
DNA: Deoxyribonucleic acid
DPWG: Royal Dutch Pharmacogenetics Working Group
FIBSER: Frequency, Intensity, Burden of Side Effect Rating
GCP: Good Clinical Practice
GP: General practitioner/general practice
HREC: Human Research Ethics Committee
ICER: Incremental cost-effectiveness ratio
MARS-5: Mediation Adherence Report Scale
MBS: Medicare Benefits Schedule
MDD: Major depressive disorder
MRFF: Medical Research Future Fund
PBS: Pharmaceutical Benefits Scheme
PGx: Pharmacogenomics
PHQ-9: Patient Health Questionnaire 9
PRESIDE: PhaRmacogEnomicS In DEpression
PRN: Pro Re Nata (as needed)
QALY: Quality-adjusted life year
RCT: Randomised control trial
SAP: Statistical analysis plan
SD: Standard deviation
SNRIs: Serotonin-noradrenaline reuptake inhibitors
SPIRIT: Standard Protocol Items: Recommendations for Interventional Trials
SSRIs: Selective serotonin reuptake inhibitors
TCA: Tricyclic antidepressants
UOML: University of Melbourne
